# The sea cucumber *Holothurialineata* Ludwig, 1875 (Holothuroidea, Aspidochirotida, Holothuriidae) re-described from the newly found type

**DOI:** 10.3897/zookeys.836.29932

**Published:** 2019-04-08

**Authors:** Yves Samyn, Claude Massin, Didier Vandenspiegel

**Affiliations:** 1 Royal Belgian Institute of Natural Sciences, Vautierstraat 29, B-1000 Brussels, Belgium Royal Belgian Institute of Natural Sciences Brussels Belgium; 2 Royal Museum for Central Africa, Leuvensesteenweg 13, B-3080 Tervuren, Belgium Royal Museum for Central Africa Tervuren Belgium

**Keywords:** Biodiversity, synonymy, taxonomy

## Abstract

A re-description of the little-known holothurian species Holothuria (Lessonothuria) lineata Ludwig, 1875 is given. It is based on the single recovered type specimen and an individual recently collected on Glorioso Islands, near Madagascar. A key to separate three closely related and commonly confused species, i.e., Holothuria (Lessonothuria) pardalis Selenka, 1867, Holothuria (Lessenothuria) verrucosa Selenka, 1867 and Holothuria (Lessonothuria) insignis Ludwig, 1875, is presented.

## Introduction

Currently approximately 150 species are recognised within the genus *Holothuria* Linnaeus, 1767 (Samyn et al. 2005). However, this is a considerable underestimation of the real number of species in this mega-diverse genus in which up to 200 species have been estimated (Honey and Solis-Marin 2018). Unfortunately, many of the recognised species are so poorly described that identification of new material remains problematic. Holothuria (Lessonothuria) lineata Ludwig, 1875 is such a case. Even though this species has been cited more than 30 times in the literature, an illustration of the ossicle assemblage is present only in the original description (Ludwig 1875). A colour picture of the external appearance of H. (L.) lineata is given only in a single recent publication (Mulochau and Conand 2008). Ludwig’s (1875) drawings of the ossicle assemblage, albeit of a good quality, do not meet today’s standard and thus require a re-description.

Study of the lectotype (designated by Rowe, in Rowe and Gates 1995: 291) and an additional voucher specimen from Glorioso Islands allowed us to define the boundaries of this species much more clearly. We consider H. (L.) lineata as valid species (see also Rowe 1989, Rowe and Gates 1995), contrary to Panning (1935) and Clark (1946) who considered it to be a junior subjective synonym of *Holothuriapardalis* Selenka, 1867.

## Materials and methods

Ossicles were removed from various tissues (tentacles, dorsal and ventral body wall, dorsal papillae, and ventral tube feet) of the lectotype and the specimen from Glorioso Islands in household bleach and were observed with light and scanning electron microscopy (*SEM*) (Samyn et al. 2006; 2007). For light microscopy, permanent slides are deposited in the collection of the Royal Belgian Institute of Natural Sciences (RBINS) (I.G. 30872/HOL.1735/1-7). For SEM, samples were dried and mounted on aluminium stubs, coated with gold in a sputter coater, and observed with a JEOL JSM-5400LV. The specimen of *Holothurialineata* from Glorioso Islands has been deposited in the collection of the RBINS (I.G. 30872/HOL.1735). The lectotype remained in the collection of the Zoological Museum Hamburg (ZMH E. 2585) .

## Taxonomy

### Holthuria (Lessonothuria) lineata

Taxon classificationAnimaliaHolothuriidaHolothuriidae

Ludwig, 1875

**Figure 1.**
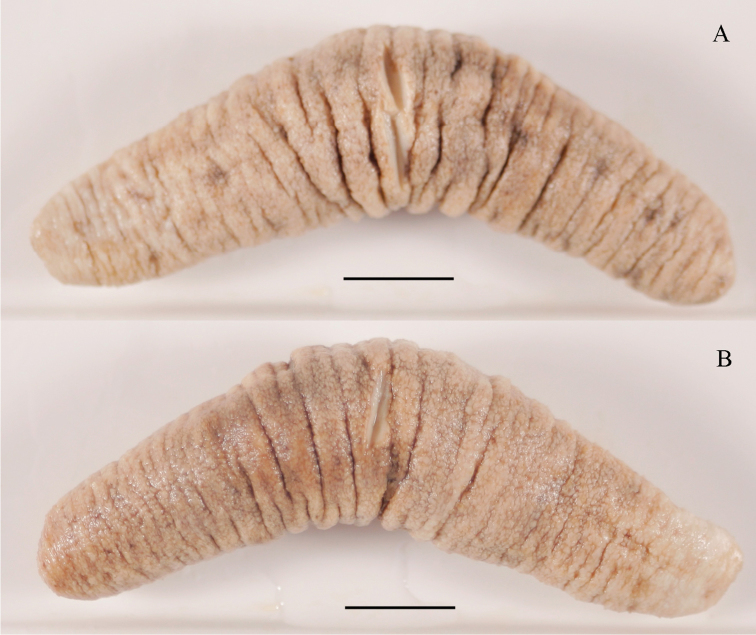
Holothuria (Lessonothuria) lineata Ludwig, 1875. **A** Dorsal view of the lectotype (ZMH E. 2585) **B** ventral view of the lectotype (ZMH E. 2585). Scale bars: 10 mm.

**Figure 2.**
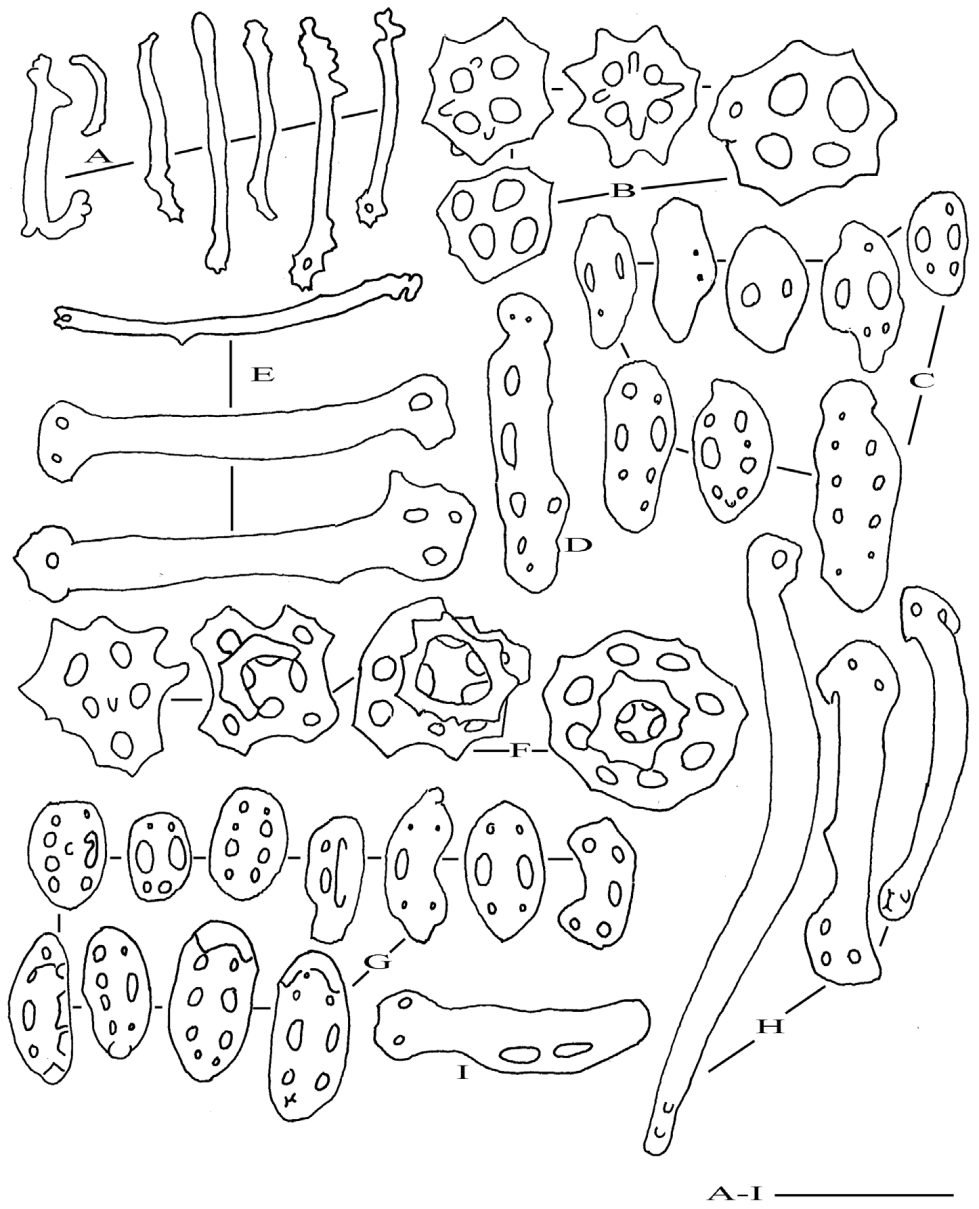
Holothuria (Lessonothuria) lineata Ludwig, 1875 (ZMH E. 2585, lectotype). **A** Rods of tentacles **B** tables of ventral body wall and ventral tube feet **C** buttons of ventral body wall and ventral tube feet **D** large button of ventral body wall **E** perforated rods of ventral tube feet **F** tables of dorsal body wall and dorsal papillae **G** buttons of dorsal body wall and dorsal papillae **H** perforated rods of dorsal papillae **I** Rod-shaped button of dorsal papillae. Scale bar: 50 µm.

**Figure 3.**
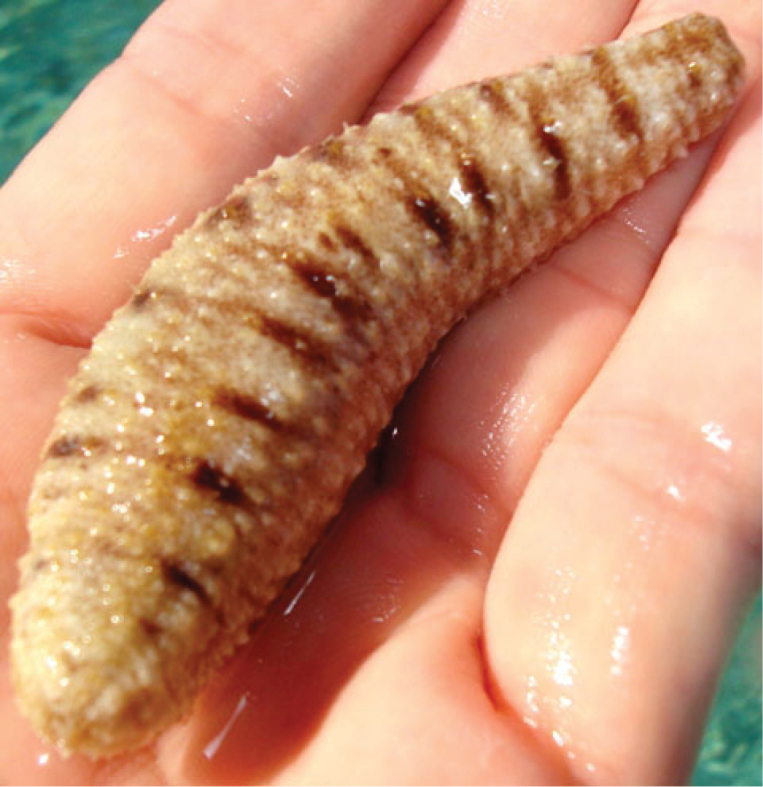
Holothuria (Lessonothuria) lineata Ludwig, 1875. Dorsal view of the specimen collected from Glorioso Islands; photograph by T Mulochau.

**Figure 4.**
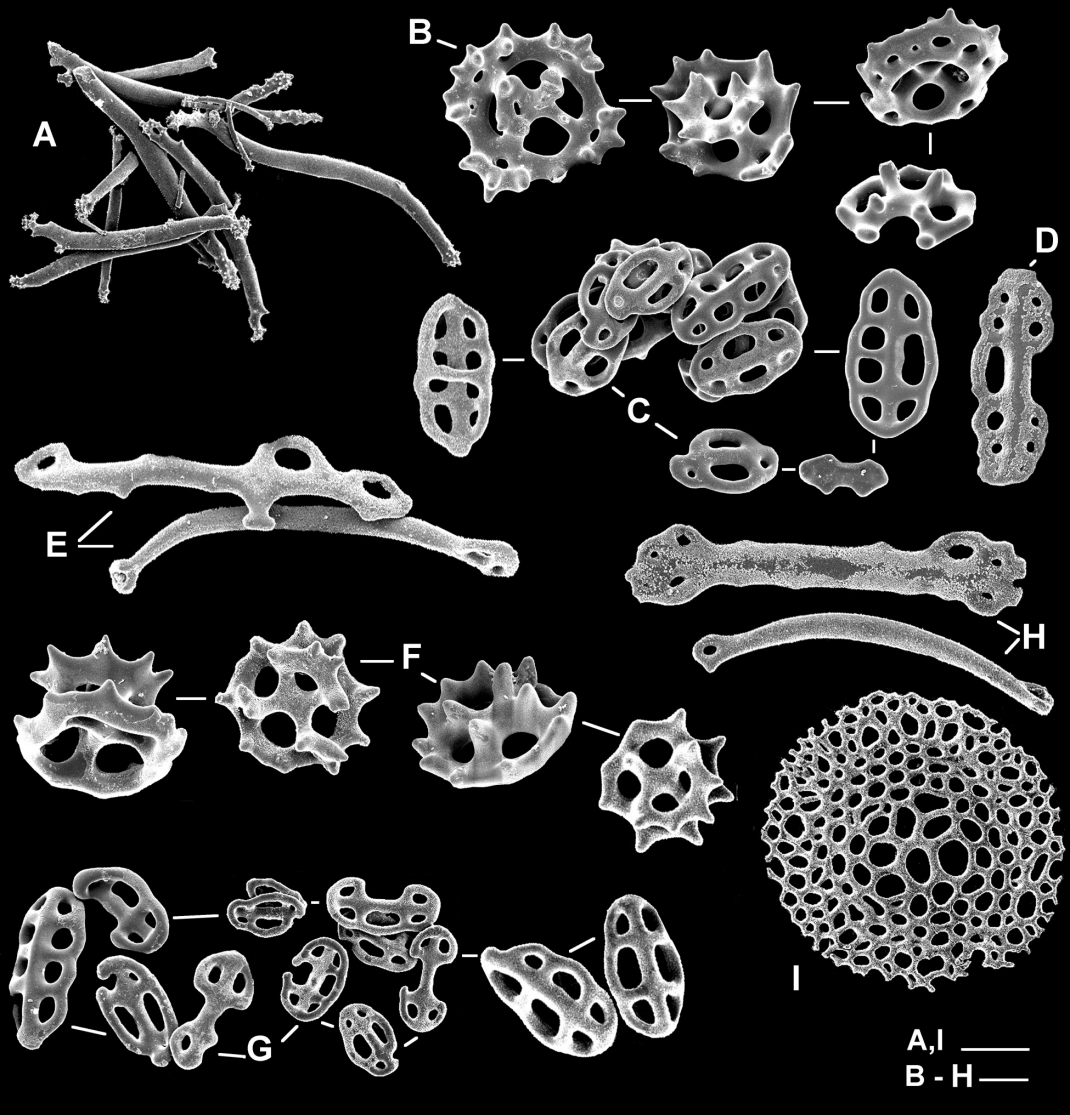
Holothuria (Lessonothuria) lineata Ludwig, 1875 (IRSNB I.G. 30872/HOL.1735). **A** Rods of tentacles **B** tables of ventral body wall and ventral tube feet **C** buttons of ventral body wall and ventral tube feet **D** rod-shaped button of ventral body wall **E** perforated rods of dorsal papillae **F** tables of dorsal body wall and dorsal papillae **G** buttons of dorsal body wall and dorsal papillae **H** perforated rods of ventral tube feet **I** end plate of ventral tube feet. Scale bars: 50 µm (**A, I**) , 20µm (**B–H**).

Figs 1A, B ; 2 ; 3A–J ; 4A–I

Holothuria lineata Ludwig, 1875: 103, pl. 2, fig. 42a-e; Ludwig 1880: 7; Ludwig 1882: 136; Ludwig 1883: 170; Bell 1884: 152; Lampert 1885: 63, fig. 26; Théel 1886: 225; Bell 1887: 140; Fisher 1907: 664; Pearson 1910: 179; Mitsukuri 1912: 118 (cited as a junior subjective synonym of H.pardalis); Clark 1925: 103; Panning 1935: 3 (cited as a junior subjective synonym of H.pardalis); Clark 1946: 437 (cited as a junior subjective synonym of H.pardalis); Sastry 2005: 105 (H.lineata; Bell 1887 non H.lineataLudwig 1875, cited as a synonym of H.pardalis); Sastry 2007: 222 (H.lineata ; Bell 1887 non H.lineataLudwig 1875, cited as a synonym of H.pardalis).
l ? Holothurialineata; Mulochau and Conand 2008: 36, fig. 3i.
Holothuria (Lessonothuria) lineata ; Rowe 1989: 282; Marsh et al. 1993: 64; Marsh 1994: 10; Rowe and Gates 1995: 291; Marsh 2000: 26; Lane et al. 2000: 488; Samyn 2003: 39; Paulay 2003: 578; Thandar 2008: 53, fig. 20A–K.
Holothuria (Lessonothuria) pardalis ; Liao and Clark, A.M., 1995: 438; Liao 1997: 105; Massin 1999: 25, figs 18a–j, 19 (non Holothuriapardalis Selenka, 1867).
Holothuria cf. pardalis ; Mulochau and Conand 2008: 36, fig. 3j.
? Labidodemaspunctulatum Haacke, 1880: 47.

#### Type material.

Lectotype *H.lineata*ZMH E. 2585 Bowen (Queensland, Australia), collection date and depth unknown, A Dietrich leg., lectotype (University of Hamburg, Zoological Museum Hamburg); formerly MG 9942 (Museum Godeffroy, Hamburg). ***Other type material***: 18 specimens according to Ludwig (1875); none recovered. ***Other material***: Glorioso Islands, 26.iv.2008, 1 m depth, Th. Mulochau leg., RBINS I.G.30872/HOL.1735 (one specimen of *H.lineata*); Indonesia, S.W. Sulawesi, 5 m depth, C Massin leg., 23.ix.1994, Pulau, Barang-Lompo, RBINS I.G. 28251/HOL.104).

#### Type locality.

Bowen (Queensland, Australia) (Ludwig, 1875).

#### Description of ZMH E. 2585, lectotype from Bowen, Australia.

(Fig. 1A, B) Specimen well preserved, poorly relaxed, partly eviscerated (part of gut missing). Body form cylindrical with extremities moderately fusiform. Length 62 mm; anterior and posterior width 7 mm; mid-body width 14 mm. Mouth and anus terminal. Colour of dorsal and ventral body wall yellow to beige irregularly marbled with brown; narrow longitudinal line along the dorsal ambulacrae clearly visible. Body wall slightly rough to the touch, ca. 1 mm thick. Position of ventral and dorsal tube feet and/or papillae difficult to determine due to poor relaxation, but tube feet appear more numerous on ventral than on dorsal surface; distribution seemingly uniform over total surface. No papillae or other appendages observed around the anus. Number of tentacles, Polian vesicles, stone canals, and shape of the calcareous ring could not be determined without causing irreversible damage to the lectotype. No Cuvierian tubules observed.

Ossicles of tentacles (from tips only, as due to contraction of specimen, shafts were not accessible) comprise few straight or slightly curved rods, 25–110 µm long with spiny extremities, sometimes perforated (Figure 2A). Ossicles of ventral and dorsal body wall, ventral tube feet, and dorsal papillae comprise tables, buttons, and rods. Tables of ventral body wall and tube feet very low, nearly always reduced to the disc, with four reduced pillars; disc 38–55 µm across, perforated by 4–5 holes; edge of disc with large blunt spines (Figure 2B). Buttons of ventral body wall and ventral tube feet generally smooth, 30–60 µm long, with 1–5 pairs of holes (Figure 2C); holes very large or nearly fully obliterated; irregular buttons numerous, some being intermediary between buttons and rods (Figure 2D). Rods of ventral tube feet wide, slightly curved, 100–150 µm long, with perforated extremities (Figure 2E). Same type of ossicles in dorsal body wall and dorsal papillae, but tables slightly larger dorsally than ventrally, 50–63 µm across (Figure 2F); spire very low ending in a partial crown of spines, sometimes fully developed bearing eight spines; edge of disc spiny; disc perforated by four central holes and eight peripheral holes. Buttons 40–65 µm long, with 3–5 pairs of holes; surface of buttons slightly knobbed; holes very large or nearly obliterated (Figure 2G). Rods of dorsal papillae 100–200 µm long, smooth with perforated extremities (Figure 2H). Intermediate form between rods and buttons very rare (Figure 2I). No ossicles observed in longitudinal muscles, cloaca, digestive tract, and respiratory trees.

#### Description of RBINS I.G. 30872/HOL.1735, non-type material from Glorioso Islands.

(Fig. 3) Single specimen well preserved, poorly relaxed. Body form cylindrical, slightly tapering at both extremities. Length 53 mm; anterior width 20 mm; posterior width 22 mm. Mouth and anus terminal. Mouth surrounded by a circle of white papillae. Colour of body wall beige-brown dorsally and beige ventrally; complete body surface speckled with minute brown dots; laterally and ventrally some transversal brown lines distinguishable; dorsal surface with conspicuous longitudinal lines along the ambulacrae and with brown blotches. Body wall soft to the touch, ca. 1.5 mm thick. Tube feet large, cylindrical, yellow with a large sucker; spread all over the ventral and dorsal surface, without alignment, more densely crowded ventrally than dorsally. Number of tentacles could not be determined without causing irreversible damage to the specimen. Calcareous ring white, extremely narrow (barely visible with the naked eye). Longitudinal muscles huge, bifid, cylindrical (3.2–5.3 mm across). Number of Polian vesicles and stone canals could not be determined. Cuvierian tubules not observed. Digestive tract full of white calcareous sand with large pieces (2 × 4 mm) of *Halimeda* sp.

Ossicles of tentacles (mainly from the shafts) comprise rods only, 150–260 µm long, smooth, perforated or slightly branched at extremities (Figure 4A). Ventral and dorsal body wall hold tables and buttons (Figure 4B, C, F, G). Ventrally, buttons 40–60 µm long, with 1–4 pairs of holes, mostly smooth; tables with spire low or completely reduced to the disc, 45–55 µm across; table discs perforated by four large central holes and 0–8 small peripheral holes; rim of disc with strong spines. Dorsally, buttons more irregular than those of ventral body wall, some with only a single row of holes; tables similar to those of ventral body wall. Ventral tube feet present buttons, tables, rods, and a single-piece end-plate of 100–200 µm across (Figure 4I). Dorsal papillae devoid of perforated plates; but with numerous, slender, 110–200 µm long rods; tables in shape and size largely as in body wall, 30–50 µm across; and irregular buttons, 35–60 µm long, perforated by 1–4 pairs of holes. Ventral tube feet with the same ossicle assemblage as dorsal papillae (Figure 4E) but, slender rods scarcer, and perforated plates, 140–160 µm long, perforated by two rows of holes, present, surrounding single-pieced end-plate of 350–400 µm across; buttons 35–60 µm long, smooth or slightly knobbed; most with two rows of holes, but many irregular ones also; buttons reduced to one row of holes very rare. No ossicles observed in longitudinal muscles, cloaca, digestive tract, and respiratory trees.

#### Distribution.

Hawaiian Ids (USA) (Fisher, 1907), Johnston Is. (USA), Mariana Ids (Guam, USA) (Paulay, 2003); Australia (NE, SE, NW, and N coasts, QLD, Thursday Is, NSW, WA, NT, Norfolk Is, Lord Howe Is., Montebello Islands, Ashmore & Cartier Islands, Tasman Sea) (Rowe 1989; Rowe and Gates 1995; Marsh 2000), Andaman Islands (Bell 1887); Sulawesi (Indonesia) (Massin 1999), Japan (Mitsukuri 1912); China, South China Sea (Liao and Clark 1995; Lane et al. 2000), Philippines, Borneo, Cocos (Keeling) Islands (Marsh 1994), Kerimba Archipelago (Mozambique) (Pearson 1910), Red Sea (Ludwig 1880), Mauritius (Ludwig 1883), Glorioso Islands (Mulochau and Conand 2008; this work), South Africa (Thandar 2008).

#### Discussion.

The two examined specimens are very similar, except for the size of the rods of the tentacles. This is because the ossicles isolated from the tentacles of the type specimen originate from the tentacle shaft, whereas those removed from the non-type specimen originate from the tentacle tip. According to numerous observations the length of tentacle rods diminishes from the base to the tip in many holothurians (e.g., Cherbonnier and Féral 1984; Cherbonnier 1988; Massin 1999).

Redescription of Holothuria (L.) lineata based on the morphological study of a specimen from Glorioso Islands and on the lectotype specimen from Bowen (Queensland, Australia) revealed that *H.lineata* is a distinct and well-diagnosed taxon, despite earlier claims (Panning 1935; Clark 1946) to consider it as a junior subjective synonym of *H.pardalis*. Moreover, *H.pardalis* (and thus *in se H.lineata*) is often confused with closely related species such as *H.verrucosa* Selenka, 1867 and *H.insignis*Ludwig 1875. Therefore a key is presented here to show the interspecific differences between these species.

*Holothuriaverrucosa* is characterised by fully developed tables with numerous (more than eight) peripheral holes and with the edge of the disk bearing numerous minutes spines (Cherbonnier 1980, 1988; Liao and Clark A.M., 1995; Samyn 2003) versus reduced tables in *H.lineata, H.insignis*, and *H.pardalis. Holothuriaverrucosa* is also characterised by the presence of 24–30 tentacles versIus 18–20 for the three other species. The ossicle assemblage of the tube feet of *H.pardalis* are characterised by massive curved rods with 1–3 perforations at the extremities versus slender curved rods with 2–7 perforations at the extremities for the three other species. *Holothuriainsignis* differs from the three other species by a majority of buttons (or pseudo buttons) being reduced to one row of perforations (see Ludwig 1875; Panning 1951, Liao and Clark 1995).

### The key below allows separation of *Holothurialineata* from the three most similar species

**Table:** 

1	24–28 tentacles, tables fully developed with up to eight peripheral perforations	*** Holothuria verrucosa ***
–	18–20 tentacles, tables reduced with no or low spire and few peripheral perforations	**2**
2	Majority of buttons with one row of holes	*** Holothuria insignis ***
–	Majority of buttons with two rows of holes	**3**
3	Length of body up to 12 cm; rods of tube feet massive, curved, with 1–3 distal perforations; perforated plates of dorsal tube feet with 3–4 rows of holes	*** H. pardalis ***
–	Length of body up to 6 cm; rods of tube feet slender, only slightly curved, with 2–7 holes at the extremities, perforated plates of dorsal tube feet with two rows of holes	*** H. lineata ***

## Conclusion

*Holothurialineata* has often been confused with other species, notably with *H.pardalis* of which it was long time considered a junior subjective synonym. In fact, Rowe (in Rowe and Gates 1995) demonstrated that the type series of *H.pardalis* contains six specimens that need to be referred to *H.lineata*. It can thus be expected that the confusion between *H.pardalis* and *H.lineata* will also exist in the literature. Such is, for instance, the case in Rowe (1985) and Massin (1999) where the *H.pardalis* specimens should be referred to *H.lineata*.

As *H.lineata* is distinctly smaller than *H.pardalis* one could argue that the former is but a juvenile of the latter. This reasoning is, however, not upheld by the ossicle assemblage of the two species.

This confusion between species also makes that the distribution of *H.lineata* and its related species largely unknown. We expect that the present re-description will help to unveil the true identity of previously and potentially newly collected specimens in this group and as such also will reveal the actual distribution of the various species.

## Supplementary Material

XML Treatment for Holthuria (Lessonothuria) lineata
